# Converging Minds: EEG Synchrony During Communication About Moral Decision-Making in Dyadic Interactions

**DOI:** 10.3390/s25134239

**Published:** 2025-07-07

**Authors:** Roberta A. Allegretta, Katia Rovelli, Michela Balconi

**Affiliations:** 1 International Research Center for Cognitive Applied Neuroscience (IrcCAN), Università Cattolica del Sacro Cuore, Largo Gemelli 1, 20123 Milan, Italy; katia.rovelli@unicatt.it (K.R.); michela.balconi@unicatt.it (M.B.); 2 Research Unit in Affective and Social Neuroscience, Department of Psychology, Università Cattolica del Sacro Cuore, Largo Gemelli 1, 20123 Milan, Italy

**Keywords:** moral decision-making, delta band, hyperscanning, EEG, affective reasoning, cognitive reasoning

## Abstract

Communication about moral decision-making involves complex emotional and cognitive processes, especially in critical situations. This study adopted a hyperscanning paradigm to explore neural convergence during moral negotiation. Twenty-six healthy young adults (mean age = 23.59 years; 16 women, 10 men), with no neurological or psychiatric conditions, were paired into 13 same-gender dyads at the Università Cattolica del Sacro Cuore. Each dyad discussed a medical moral dilemma while their electrophysiological (EEG) activity was simultaneously recorded. Participants were first categorized according to their Dominant Reasoning Profile (DRP) (cognitive or affective), and subsequently convergence in DRP within the dyads was established. EEG band dissimilarities within each dyad were analyzed across frontal, temporo-central, and parieto-occipital regions. The results revealed significantly greater dissimilarity in frontal delta-band activity compared to parieto-occipital areas, regardless of the dyad’s DRP. Such results might suggest different emotional and motivational reactions between the two individuals, reflecting a broader gap in how the moral decision-making process was interpreted and internalized by each member, despite their DRP. The EEG hyperscanning paradigm proves useful in the study and understanding of the neural mechanisms involved in social interaction about morally sensitive decisions and provides novel insights into dyadic brain dynamics.

## 1. Introduction

Moral dilemmas have long captured the interest of philosophers and psychologists, often portrayed as decisions made in solitude by a lone agent confronting a difficult choice. Yet in everyday life, moral decisions rarely occur in isolation. From group deliberations in juries to informal discussions among peers, our moral choices are frequently formed, challenged, and reshaped through interaction with others by dialogue, group norms, and shared values [1].

A moral dilemma is typically presented as a brief narrative describing a situation marked by a moral conflict. This kind of conflict arises when an individual is torn between opposing courses of action, each supported by competing moral justifications. It involves the recognition that the available options are mutually exclusive, and that each choice leads to distinct, and often ethically significant, consequences [2]. Moral conflict can manifest in various forms. It may involve (i) a struggle between personal interests and widely accepted moral norms; (ii) a clash between competing duties or obligations; (iii) a confrontation between sets of values that appear incommensurable; or even (iv) internal conflict stemming from the application of a single moral principle that yields contradictory prescriptions. In all cases, the individual is confronted with the challenge of navigating a decision space shaped by competing normative demands [2].

Therefore, it is clear that moral decision-making may be accompanied by heightened emotional arousal. It is thus unsurprising that individuals tend to seek dialogue with others when confronted with such dilemmas, rather than relying solely on solitary reflection. This tendency is likely to be even more pronounced in situations where decision-making involves complex moral considerations [3].

To this purpose, the Dual Process Hypothesis of Moral Judgment (DPHMJ) offers a framework for understanding the variability in human moral judgments [4]; according to this model, moral reasoning arises from the interaction between two systems: a cognitive, top–down process driven by deliberate reasoning and a bottom–up, emotionally based process triggered by affective responses. Findings from cognitive neuroscience further support this view, showing that brain regions involved in both cognition and emotion contribute to moral reasoning [5]. In general, studies have shown that individuals may have different information processing approaches based on their need for affect (NFA) or need for cognition (NFC); notably, people with high NFA often rely on emotional information when forming attitudes and regulating their behavior [6,7]. On the other hand, those with high NFC are more likely to base their evaluations on careful analysis and pay close attention to the attributes of the object they are assessing.

What stands out, however, is the relative scarcity of research examining how people engage in moral reasoning through social interaction, despite the abundance of studies focusing on moral dilemma resolution as an individual cognitive process [3]. For this reason, neuroscience can contribute significantly to the study of social interaction about moral decision-making by shedding light on underlying mental processes that are not directly observable. Through techniques that monitor real-time neural activity, it offers valuable insights into cognitive mechanisms that function independently of language and are often difficult to articulate through verbal expression [8]. In addition, to capture the complexity of real-time interpersonal dynamics, neuroscience has progressively embraced the “hyperscanning” paradigm, signaling a move beyond conventional single-brain studies toward what is now known as “two-person neuroscience.” This methodology allows for the simultaneous measurement of neural activity in multiple individuals during live interaction, providing important insights into the reciprocal and co-constructed nature of social cognitive processes [9].

Studies exploring the neural mechanisms underlying successful communication and reciprocity in social interactions reveal how brain activity synchronizes between individuals and influences cooperative behavior. Notably, a study investigating the neural correlates of group decision-making in social dilemmas using hyperscanning [10] showed that group decisions elicited significantly higher inter-brain synchrony in the inferior frontal gyrus (IFG) and dorsolateral prefrontal cortex (DLPFC), regions associated with cognitive control and social coordination. Instead, a recent study [11] using a multimodal hyperscanning paradigm found, during a negotiation process, reduced frontal activation and a shift toward the temporoparietal junction (TPJ), which is linked to perspective-taking. Additionally, EEG findings indicated heightened delta, theta, and alpha activity in the frontal region, reflecting emotional and motivational engagement. Meanwhile, beta and gamma activity were more pronounced in temporo-central and parieto-occipital regions, signaling cognitive integration and perspective-taking. These results highlight negotiation as an emotionally and cognitively demanding process, requiring both strategic adaptation and interpersonal alignment to reach a shared agreement.

Another study [1] employing functional Near-Infrared Spectroscopy (fNIRS) to investigate the neurophysiological correlates of moral decision-making within socially and professionally relevant contexts found distinct patterns of cortical activation depending on the nature of the moral choice and the context. Specifically, increased activation in the DLPFC and ventromedial prefrontal cortices (VMPFCs) was observed during fair decisions in personally relevant conditions, reflecting cognitive control and emotional valuation. In contrast, heightened activation in the anterior temporal cortex, particularly the superior temporal sulcus (STS), was associated with unfair offers in social contexts, implicating processes of empathy and mentalizing. These results underscore the complex interplay between emotional and cognitive systems in interpersonal moral reasoning and point to specific neuroanatomical substrates supporting fairness-related processing in dyadic interactions. Taken together, these studies suggest that effective communication and successful social cooperation rely on a balance between emotional, cognitive, and predictive processes, with neural synchronization facilitating understanding and mentalizing mechanisms guiding reciprocal interactions.

Interestingly, moral reasoning engages several overlapping brain regions, including the DLPFC, orbitofrontal cortex (OFC), VMPFC, anterior cingulate cortex (ACC), precuneus, TPJ, parietal lobe, and superior medial prefrontal cortex [1,12]. Notably, the VMPFC is crucial for modulating emotional responses associated with moral choices and personal values. The OFC contributes to the assessment of morally salient information, particularly in relation to feelings of guilt, while the TPJ and precuneus are key to perspective-taking and understanding moral intentions in others.

However, most of these studies used functional imaging techniques, such as functional magnetic resonance imaging (fMRI), that, despite its high spatial resolution, can limit a naturalistic interaction. Conversely, electroencephalography (EEG) is a valuable tool due to its high temporal resolution, cost-effectiveness, and ease of use, making it ideal for tracking neural activity over time. By analyzing EEG frequency bands (delta, theta, alpha, beta, and gamma), along with their functional relevance, scalp distribution, and hemispheric lateralization, EEG can be employed to investigate brain activity associated with the cognitive and emotional processes involved in social interactions.

According to existing research, low-frequency EEG bands (delta and theta) are typically associated with emotional processes [13,14,15]. Specifically, delta activity is strongly related to emotional regulation and response planning in challenging situations, particularly in the temporo-central regions. Theta activity, predominantly found in the mid-frontal area, is involved in attention and memory, and when observed over parietal regions, is linked to problem-solving, especially in the context of social decision-making. In contrast, high-frequency bands (alpha, beta, and gamma) are generally associated with cognitive effort, information processing, engagement, and mechanisms of focused attention.

However, to the best of our knowledge, no study has focused on social interactions about moral decision-making through an EEG hyperscanning paradigm and specifically investigated how interpersonal neural dissimilarity emerges. Therefore, the primary objective of the present study is to investigate whether and how patterns of neural divergence between individuals arise during real-time communicative exchange involving a moral dilemma, using an EEG hyperscanning approach, by grouping dyads according to their moral reasoning propensity in solving a moral dilemma (i.e., cognitive or affective). Specifically, participants engaged in a naturalistic social interaction that involved discussing a moral decision within a realistic medical scenario. After reading the scenario, both participants independently chose what they believed to be the most appropriate resolution to the moral dilemma from a list of predefined options. Afterwards, both discussed their chosen solution, trying to negotiate a shared decision. Throughout the entire interaction, EEG data were continuously recorded from both participants. This study should be considered a pilot investigation aimed at assessing the feasibility and methodological validity of applying EEG hyperscanning to the study of moral decision-making during dyadic interaction. Indeed, this study’s objective was to preliminarily test the ecological viability of the experimental paradigm and identify meaningful neural markers of interpersonal dissimilarity. Therefore, given this theoretical background, we aim to find a greater dissimilarity in frontal areas in low-frequency bands (and specifically delta, which is strongly related to emotional regulation and response planning) compared to the temporo-central area, reflecting the differential involvement of these areas in processing moral versus communicative aspects of interaction. Moreover, we expect to find this greater inter-brain dissimilarity despite the moral reasoning propensity of the dyads being congruent or divergent; indeed, frontal areas are critically involved in integrating both emotional and cognitive components of moral decision-making, as well as regulating affective responses. These processes are highly individual and context-dependent, likely leading to greater variability in neural activation patterns. Therefore, higher interindividual dissimilarity is expected in frontal regions, reflecting the diverse strategies participants may adopt when engaging in morally charged interpersonal discussions

## 2. Method

### 2.1. Sample

The sample consisted of 26 right-handed young adults (16 women, 10 men; mean age = 23.59 years, SD = 2.00), recruited through convenience sampling without probabilistic criteria. Participants were randomly designated to complementary roles within the experimental dyad (designated as member A and member B) and paired into 13 same-gender dyads (8 female, 5 male) (Table 1). Prior familiarity between dyad members was rigorously excluded to prevent relational bias and ensure the independence of interpersonal dynamics. The task design did not involve distinct experimental groups or conditions. All dyads engaged in the same two-step Moral Deliberation Task (MDT), and no differential treatment or manipulation was applied across participants. However, in line with a single-blind study design, participants were not informed about the study’s specific hypotheses and remained unaware of their partner’s reasoning profile throughout the interaction, thereby reducing bias and preserving the integrity of interpersonal deliberation.

Eligibility was restricted to individuals with normal or corrected-to-normal vision and no record of psychiatric or neurological illness. Participants presenting with clinically significant depressive symptoms, memory dysfunctions, cognitive impairments, or undergoing treatment with psychoactive substances were excluded from enrollment. No monetary or material compensation was provided for participation.

All procedures conformed to international ethical research standards. This study received formal authorization from the Ethics Committee of the Department of Psychology, Università Cattolica del Sacro Cuore, Milan (approval protocol 125/24; project title: Valutare il Decision-Making: consapevolezza e metacognizione decisionale; approval date: 23 July 2024). This research was conducted in full compliance with the ethical principles of the Declaration of Helsinki (2013) and the European General Data Protection Regulation (Reg. EU 2016/679) [16].

### 2.2. Experimental Procedure

**Figure 1:**
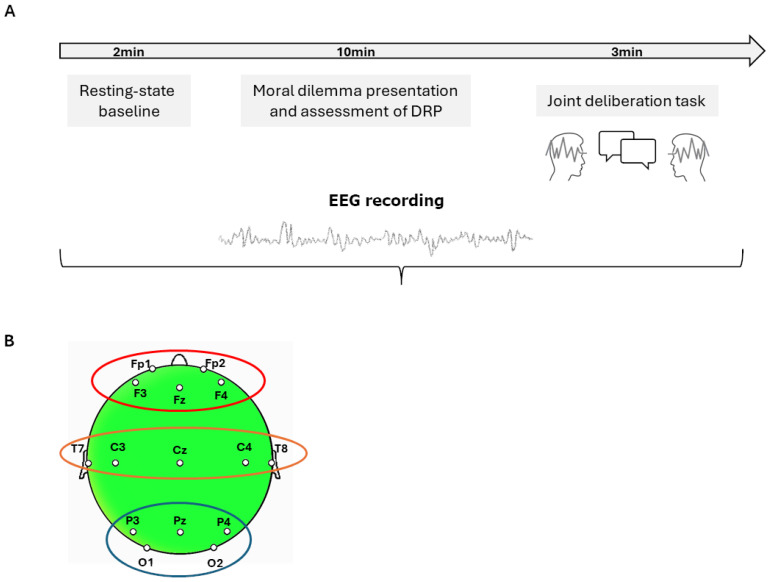
(**A**) Experimental procedure of the Moral Deliberation Task (MDT). Participants were seated face-to-face and underwent simultaneous EEG hyperscanning during a two-phase task: (1) individual assessment of Dominant Reasoning Profile (DRP; affective vs. cognitive) in response to a medical moral dilemma and (2) dyadic negotiation to reach a shared decision. (**B**) EEG recordings were conducted using 15 selected electrodes per subject, positioned according to the international 10–20 system. For the purposes of statistical analysis, data were grouped into three anatomically defined regions of interest (ROIs): frontal (Fp1, Fp2, F3, F4), temporo-central (T7, T8, C3, C4), and parieto-occipital (P3, P4, O1, O2).

Prior to the onset of the experimental protocol, all participants received standardized verbal and written instructions to ensure comprehension and compliance with procedural directives. The experiment was conducted in a sound-attenuated room and lasted approximately 30 min (Figure 1A,B).

Participants were seated face-to-face to facilitate direct verbal interaction while minimizing environmental interference.

Simultaneous EEG recordings were acquired from both members of each dyad using a hyperscanning paradigm. Neural data were collected during two distinct phases: (i) a resting-state baseline (duration: 120 s) and (ii) an MDT-related phase involving interactive moral deliberation. Specifically, the MDT phase was divided into two steps: Step 1, which aimed to assess participants’ dominant reasoning propensity (i.e., cognitive or affective) through four statements, and Step 2, where participants were asked to engage in a negotiation and come to a unanimous agreement, choosing one of the four statements they had previously assessed.

Throughout the session, participants were instructed to maintain a stable posture, articulate verbalizations clearly, and alternate turns to prevent speech overlap. A full-session audiovisual recording was employed to annotate and control significant non-verbal behavioral deviations (e.g., facial expressions, postural shifts) potentially influencing interpersonal dynamics.

#### 2.2.1. Moral Deliberation Task (MDT)—Step 1

The experimental paradigm presented a realistic moral dilemma set in a COVID-19 emergency, where participants assumed the role of physicians forced to prioritize one of two clinically similar elderly patients for scarce treatment resources. Although both patients were equal in age and medical risk, they differed in personal background; one had hypertension and a supportive daughter with a chronic illness, and the other had diabetes but no contextual details. These narrative differences were designed to elicit emotional engagement while preserving clinical equivalence.

After being presented with the scenario, participants were invited to consider the physician’s decision-making process and respond to the following question: “If you were the physician, to which of the two patients would you have given treatment priority?” They were then asked to indicate their level of agreement with four alternative justifications for prioritization, each corresponding to a specific type of reasoning. These justifications were designed to implicitly reflect two distinct evaluative domains without explicitly labeling them for participants. Two of the statements reflected an emotionally oriented perspective. The first justification emphasized the first patient’s dual vulnerability—his own chronic condition and the dependent care needs of his ill daughter—implying a prioritization driven by relational responsibility and empathic concern. The second emotional justification focused on the severity of the hypertensive condition, highlighting the suffering already endured by the patient and the perceived criticality of his clinical status.

The remaining two statements reflected a cognitively driven evaluative frame. One of these emphasized the second patient’s more favorable clinical prognosis, noting that the diabetic condition was associated with lower immediate risk compared to severe hypertension and therefore offered a higher likelihood of treatment success. The final justification focused on systemic efficiency, suggesting that if stabilized rapidly, the diabetic patient could be discharged sooner, thereby freeing resources for more severe cases. Participants rated each justification on a five-point Likert scale, ranging from complete disagreement (1) to complete agreement (5). No mention was made of the emotional or cognitive nature of the statements, to avoid priming or response bias. From these responses, two continuous variables were calculated for each participant. The *Affective Rating Index* (ARI) represented the mean agreement with the emotionally salient justifications, while the *Cognitive Rating Index* (CRI) captured the mean agreement with the analytically grounded justifications.

Based on the relative predominance of these two indices, each participant was categorized into a *Dominant Reasoning Profile* (DRP). Individuals who scored higher on the ARI than on the CRI were identified as affectively oriented (DRP-A), whereas those with higher CRI scores were classified as cognitively oriented (DRP-C). Participants who exhibited equivalent values on both indices, as calculated at the second decimal level, were assigned to a balanced profile (DRP-EQ).

**Figure 2:**
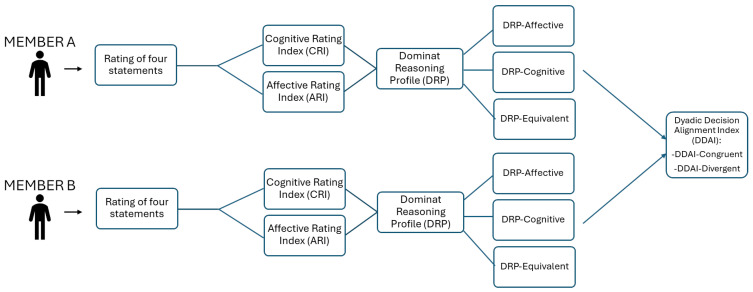
Flowchart illustrating the procedure for the classification of DDAI for each dyad.

This individual-level classification enabled the derivation of a dyadic variable termed the *Dyadic Decision Alignment Index* (DDAI), reflecting the degree of moral reasoning congruence between dyad members. Dyads in which both individuals shared the same Dominant Reasoning Profile (either DRP-A or DRP-C) were categorized as DDAI-Congruent, whereas dyads comprising members with opposing evaluative orientations were classified as DDAI-Divergent (see Figure 2).

#### 2.2.2. Moral Deliberation NEGOTIATION Task (MDT)—Step 2

Subsequent to the individual assessment, each dyad engaged in a joint deliberation phase. The participants were instructed to negotiate and reach a unanimous decision regarding which patient to prioritize, selecting one of the four justifications previously evaluated. The interaction was constrained to a maximum duration of 180 s to simulate real-world time pressure and limit excessive cognitive elaboration. During this MDT, continuous EEG data were recorded simultaneously from both members of the dyad using a hyperscanning setup. The primary objective of the MDT was to investigate whether the neurophysiological patterns associated with joint moral decision-making varied according to the alignment or divergence of moral reasoning styles within the dyad.

### 2.3. EEG Data: Acquisition and Processing

Prior to the onset of the MDT, a resting-state baseline was recorded over a 120 s interval in order to obtain reference measures of both cortical and autonomic activity under low-stimulation conditions. Electroencephalographic signals were continuously acquired throughout the baseline and task phases using a 16-channel direct current amplifier (SYNAMPS; Compumedics Neuroscan), operating in conjunction with the NEUROSCAN 4.2 acquisition suite. Electrode placement conformed to the standard international 10–20 system [17], with a total of 15 active Ag/AgCl electrodes positioned at frontal, central, temporal, parietal, and occipital sites (Fp1, Fp2, F3, F4, Fz, Cz, C3, C4, T7, T8, Pz, P3, P4, O1, O2). Linked earlobes served as the reference, and the ground electrode was placed at AFz. Horizontal eye movements and blinks were monitored via two bipolar electrooculographic (EOG) channels placed at the outer canthus and below the left eye.

Impedance for all electrodes was maintained below 5 kΩ throughout the session to ensure stable recording conditions and an optimal signal-to-noise ratio. Data were digitized at a sampling frequency of 1000 Hz, and a notch filter centered at 50 Hz was applied online to suppress power line interference. For post hoc signal processing, raw EEG traces were bandpass filtered using a zero-phase infinite impulse response (IIR) filter with cutoff frequencies set between 0.01 and 50 Hz. The resulting continuous data were then segmented into consecutive epochs of two seconds each.

Artifacts related to ocular activity, excessive muscular tension, or movement were identified and excluded through visual inspection by two independent researchers blind to the experimental conditions. Only artifact-free epochs were retained for spectral analysis. Power spectral density (PSD) estimates were computed via fast Fourier transform (FFT), employing a Hamming window function to minimize spectral leakage. The spectral resolution was fixed at 0.5 Hz, and absolute power values were extracted across the following canonical EEG frequency bands: delta (0.5–3.5 Hz) [18,19], theta (4–7.5 Hz) [20,21], alpha (8–12.5 Hz) [22,23], beta (13–30 Hz) [24,25], and gamma (30.5–50 Hz) [26].

To quantify the neural modulation associated with the MDT, task-related PSD values were normalized relative to baseline using this proportional change formula: [Normalized PSD = (PSD_MDT_ − PS_DBL_)/PS_DBL_], where PSD_MDT_ represents task-induced power and PSDBL represents baseline power.

Subsequent analyses focused on three anatomically predefined regions of interest (ROIs), each comprising functionally relevant electrode clusters: the frontal region (ROI-F: Fp1, Fp2, F3, F4), the temporo-central region (ROI-TC: T7, T8, C3, C4), and the parieto-occipital region (ROI-PO: P3, P4, O1, O2). Within each ROI, normalized PSD values were averaged across constituent channels to obtain a regional index of oscillatory activity. These indices were then used to assess frequency- and region-specific neural dynamics associated with moral reasoning under interactive conditions, relative to the resting-state baseline.

### 2.4. Data Analysis

To examine inter-brain synchronization, EEG data were analyzed at the dyadic level using Euclidean distance (Eu_Dist_) as a dissimilarity metric. This measure quantifies absolute differences in power spectral density (PSD) between the two members of each dyad and has been widely used in hyperscanning research to capture interpersonal neural divergence. EuDist was calculated for each frequency band and for each region of interest (ROI), thereby providing an inverse index of neural similarity; higher Eu_Dist_ values reflect greater neural dissimilarity, whereas lower values indicate greater inter-brain alignment. Five EEG frequency bands were examined: delta, theta, alpha, beta, and gamma. To control for potential confounds related to verbal behavior, speech durations were recorded and analyzed across dyad members to ensure comparable participation during negotiation turns. Eu_Dist_ values were calculated by computing the absolute differences in PSD between dyad members within each of three predefined ROIs: ROI-F, ROI-TC, and ROI-PO. These inter-brain distance values served as inverse indicators of neural synchrony; lower Eu_Dist_ values correspond to higher similarity in spectral power distribution between the two individuals during the task.

To control speech-related variability, mean speech durations were computed for each participant, considering only task-relevant negotiation turns. Non-negotiation utterances and pauses were excluded from this computation. Averaged speech durations and their standard deviations were examined to verify homogeneity across dyad members, ensuring comparable interaction dynamics.

For statistical analysis, a set of five repeated measures ANOVA, with DDAI (2: DDAI-Congruent; DDAI-Divergent) as a between-subject factor and ROI (3: ROI-F, ROI-TC, ROI-PO) as within-subject factor, was computed for each of the following frequency bands: delta (0.5–3.5 Hz), theta (4–7.5 Hz), alpha (8–12.5 Hz), beta (13–30 Hz), and gamma (30.5–50 Hz). When sphericity assumptions were violated, Greenhouse–Geisser correction was applied. Significant effects were followed up with Bonferroni-adjusted post hoc comparisons, and effect sizes were reported as partial eta squared (η^2^_p_). The alpha threshold for significance was set at 0.05.

Prior to the main analyses, the distributional properties of the data were assessed through skewness and kurtosis indices, and a Shapiro–Wilk test was performed to confirm approximate normality. Descriptive statistics, including condition-wise means and standard errors, were calculated to aid in interpretation. All statistical procedures were conducted using Jamovi software (version 2.6.22; The Jamovi Project, 2022).

## 3. Results

The aim of the statistical analyses was to examine inter-brain dissimilarity in EEG activity across specific frequency bands and cortical regions during dyadic moral deliberation. To this end, we computed normalized Euclidean distances for each dyad and compared spectral power differences across three regions of interest (frontal, temporo-central, parieto-occipital) and five EEG bands (delta, theta, alpha, beta, gamma). We also assessed whether dyadic alignment in the Dominant Reasoning Profile (DDAI: congruent vs. divergent) influenced inter-brain dissimilarity patterns. Below, we report the results separately by frequency band, highlighting significant effects and post hoc comparisons.

**Figure 3:**
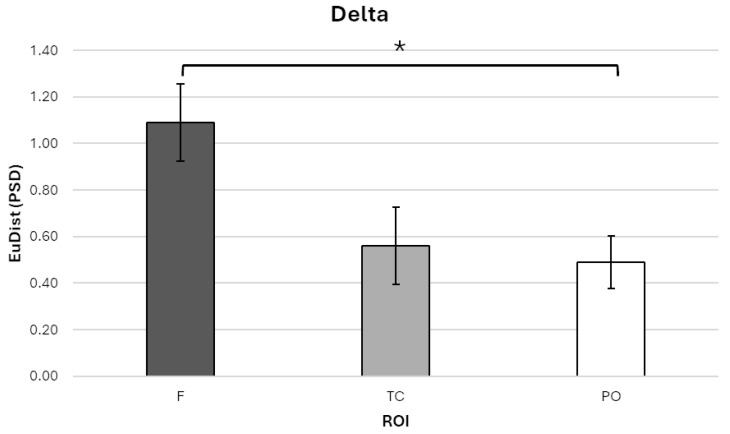
Bar graph illustrating Euclidean distance (EuDist) values between the power spectral density (PSD) in the delta frequency band across three regions of interest (ROIs): frontal (ROI-F), temporo-central (ROI-TC), and parieto-occipital (ROI-PO). Statistical analyses revealed a significant main effect of ROI, with post hoc Bonferroni-corrected comparisons indicating that EuDist was significantly higher in the ROI-F compared to the ROI-PO. Bars represent ±1 standard error and stars (*) mark statistically significant effects.

For the delta band, a significant main effect of ROI was found, F (1.24, 13.61) = 6.464, *p* = 0.019, η^2^_p_ = 0.370. Bonferroni-corrected post hoc comparisons showed that EuDist was significantly higher in the frontal ROI (ROI-F; M = 1.090, SE = 0.167, 95% CI [0.723, 1.457]) compared to the parieto-occipital ROI (ROI-PO; M = 0.489, SE = 0.112, 95% CI [0.244, 0.735]; *p* = 0.023; Figure 3).

No significant differences were found between ROI-F and the temporo-central ROI (ROI-TC; M = 0.561, SE = 0.165, 95% CI [0.197, 0.924]), nor between ROI-TC and ROI-PO.

For the theta, alpha, beta, and gamma bands, no significant main effects or interactions were observed (all *p* > 0.05) (Table 2).

These results confirm our hypothesis by indicating increased neural dissimilarity over frontal regions in the delta frequency band, independent of DDAI congruence or divergence within the dyads.

## 4. Discussion

The primary objective of the current research was to investigate similarities in neurophysiological activity within two individuals through a hyperscanning paradigm during a communicative exchange involving a moral decision-making scenario. Based on their cognitive or affective tendency in responses, the DRP for each participant was calculated. Accordingly, convergence in DRP within the dyads was established. Subsequently, based on this convergence/divergence, we aimed to examine the degree of synchrony that emerges in response to moral negotiation. Specifically, we hypothesized that low-frequency band (and specifically delta) dissimilarity would be more pronounced in frontal regions compared to other areas, irrespective of whether the dyad members shared the same Dominant Reasoning Profile (DRP).

The results supported this hypothesis; specifically, the findings suggest that the individual DRP—whether affective or cognitive—and its congruence or divergence within the dyad, exert limited influence on alignment about moral decision-making. Rather, the phenomenon appears to be more strongly associated with brain area localization in a specific frequency band, pointing to the role of distinct cortical regions in supporting interpersonal convergence during moral reasoning. This suggests that alignment in moral judgment may depend less on shared reasoning styles and more on the coordinated activation of brain areas involved in cognitive control, social cognition, and emotional regulation. These results underscore the importance of investigating the neuroanatomical substrates of dyadic moral processes, beyond individual profiles or their compatibility.

Specifically, the results revealed greater dissimilarity in delta activity over the frontal regions compared to the temporo-central area. Notably, this increased dissimilarity in the frontal region was independent of whether the dyads presented the same or different DRP.

Delta wave activity plays a fundamental role in emotional regulation processes. Specifically, fluctuations in delta band oscillations have been associated with the interpretation of both positive and negative social interactions, indicating that delta frequencies enhance attentional focus on emotionally meaningful stimuli [27]. Likewise, shifts in delta activity may reflect internal emotional states and the mechanisms involved in their regulation. These observations align with research showing increased delta activity during tasks that involve emotional processing [13]. Additionally, delta oscillations have been increasingly associated with interpersonal synchronization and empathy during social interactions. For example, a study [28] found that delta–alpha coupling observed during motor synchronization tasks reflects attention to visceral signals, which play a critical role in interpersonal engagement. This suggests that frontal delta activity may support interoceptive awareness, a key component in coordinating actions and responses within social contexts. In adult populations, other research has demonstrated that increased connectivity in the delta frequency band during face-to-face interactions is positively correlated with social attunement [29]. This enhanced connectivity appears to facilitate both communication and emotional understanding between individuals, underscoring the functional role of frontal delta oscillations in promoting effective social functioning.

Therefore, based on this evidence, we speculate that the increased delta dissimilarity observed over frontal regions might reflect divergent emotional and motivational responses between members, reflecting a greater divergence in how each individual processed and internalized the solution to the moral dilemma, despite their DRP being congruent or divergent. On the other hand, this interpretation should be complemented by considering the comparison with the temporo-central areas, and, namely, the increased synchrony observed in such areas.

In fact, the similarity in temporo-central areas may functionally reflect shared engagement in perspective-taking processes and mutual attunement, which are essential to moral reasoning and social coordination. Rather than viewing frontal dissimilarity as the only indicator of interpersonal misalignment, the temporo-central synchrony should be considered a meaningful marker of dyadic coherence. This finding aligns with prior neurofunctional evidence implicating regions such as the TPJ in understanding others’ viewpoints, interpreting moral intentions, and engaging in self-referential thought [1,12,30]. Indeed, during moral decision-making, both individuals might similarly evaluate the moral aspects of the dilemma or consider the perspectives of their counterpart, leading to convergent activation in these regions as part of their joint perspective-taking effort during the discussion.

This interpretation is supported by previous studies investigating the neural mechanisms of social interaction, communication, and reciprocity. For instance, research on speaker–listener neural coupling has shown that successful communication is associated with synchronized activity in key brain areas, including the frontal areas. These areas play a crucial role in anticipating speech and inferring the speaker’s intentions, highlighting the importance of neural alignment in achieving mutual understanding [31]. Additionally, greater inter-brain synchrony in the IFG and DLPFC during group decision-making has been linked to cognitive alignment and shared goal pursuit [10], whereas decreased frontal activation and a shift toward TPJ engagement during negotiation tasks suggest a reallocation of resources toward perspective-taking mechanisms [11].

Indeed, reciprocity in social decision-making recruits emotional and mentalizing systems. The amygdala encodes emotional valence related to cooperation or non-cooperation, while regions like the VMPFC and precuneus are involved in predicting and evaluating others’ strategies [32], with VMPFC activity reflecting accurate interpretation of reciprocal intent, and precuneus activity indicating more effortful or uncertain social inference.

In light of these findings, the increased delta-band dissimilarity we observed over frontal areas could signal a misalignment in emotional and motivational states that disrupts mutual prediction and understanding, especially at the beginning of the interaction, where participants need to negotiate. This neural divergence may reflect differing internal models or interpretations of the moral dilemma, which ultimately lead to different activation, even when both individuals shared a similar DRP. Hence, effective joint reasoning may rely not only on shared perspectives but also on synchronized affective and cognitive processing at the neural level.

Finally, the absence of significant effects in the theta, alpha, beta, and gamma bands may reflect the functional specificity of delta oscillations in capturing emotionally and motivationally salient aspects of dyadic moral negotiation. While higher-frequency bands are generally linked to cognitive control, attention, and integration processes (e.g., alpha: top–down regulation; beta/gamma: decision-making and social cognition) [33,34], these mechanisms may have been similarly recruited across participants, leading to reduced inter-dyad variability.

From a clinical perspective, these findings may help inform ethical decision-making training in healthcare settings, where professionals are frequently required to negotiate morally sensitive decisions in team-based contexts. The observed neural patterns—particularly delta-band dissimilarity in frontal regions—could serve as potential indicators of emotional misalignment during high-stakes interpersonal reasoning. Future research might explore whether such neural markers can be leveraged in clinical populations or therapeutic interventions aimed at enhancing interpersonal understanding and emotional attunement in morally complex scenarios.

While this study offers valuable insights into communicative exchange within moral decision-making contexts, several limitations should be acknowledged. First, the main limitation is the small sample size, which could reduce the statistical strength of the results. It is advised that future studies include a larger sample size to confirm these findings; moreover, future research would benefit from including a more heterogeneous sample, encompassing individuals with diverse backgrounds and experiences (e.g., medical doctors) that may influence communicative dynamics and moral reasoning. Second, to enhance the generalizability of the findings across different contexts and populations, future research should examine potential effects and variations related to gender and age. A further limitation concerns the absence of a priori statistical power calculation. Given the exploratory and pilot nature of the present study, the sample size was not determined through probabilistic estimates of statistical power. As a result, the findings, while methodologically grounded, should be interpreted with caution. Consequently, the results are not intended to be representative of the broader population and cannot be generalized beyond the specific sample studied.

Additionally, to better understand the underlying mechanisms of communicative exchange, it would be useful to investigate additional factors such as personality dimensions. Thirdly, a more holistic understanding of the neurophysiological underpinnings of communicative exchange on moral decision-making could be achieved through a multi-method approach—combining EEG frequency analysis with autonomic measures and hemodynamic responses via functional Near-Infrared Spectroscopy (fNIRS) in dyadic interactions. Moreover, one important limitation of the present study concerns the use of the Dominant Reasoning Profile (DRP), a task-specific classification developed ad hoc to capture participants’ cognitive or affective moral orientation. While theoretically grounded, this measure has not yet been formally validated, and its generalizability remains to be established. Its use was motivated by the need to reflect reasoning styles directly tied to the experimental context; however, future studies should aim to validate this construct and consider integrating standardized dispositional measures (e.g., NFA, NFC) to enhance robustness and external reliability. Finally, one methodological limitation concerns the lack of analysis regarding hemispheric lateralization and EEG microstates. Although our primary focus was on inter-brain dissimilarities in spectral power across broader cortical regions, future studies could benefit from incorporating hemispheric asymmetry indices to explore emotional valence and motivational directionality during moral negotiation. Additionally, EEG microstate analysis may offer valuable insight into the temporal dynamics of cognitive and affective processing, enabling a more fine-grained understanding of the spatiotemporal organization of brain activity. Including these parameters, alongside spectral indices, would allow for a more comprehensive investigation of the neural correlates underpinning interpersonal alignment and divergence in morally charged contexts.

## 5. Conclusions

This study should be considered a pilot investigation aimed at assessing the feasibility and methodological validity of applying EEG hyperscanning to the study of moral decision-making during dyadic interaction. Indeed, this study’s objective was to preliminarily test the ecological viability of the experimental paradigm and identify meaningful neural markers of interpersonal dissimilarity. These findings are intended to inform and refine future large-scale investigations involving more diverse populations and additional experimental conditions.

In conclusion, the findings suggest that during a social interaction involving moral content, increased delta dissimilarity over frontal regions may reflect divergent emotional and motivational processing between dyad members, regardless of whether they share or not the same DRP. In contrast, the similarity observed in temporo-central areas points to a shared engagement in perspective-taking processes, which are essential for mutual understanding and moral reasoning. Overall, these results highlight the importance of both emotional alignment and cognitive synchronization in supporting effective cooperation and joint moral decision-making.

## Figures and Tables

**Table 1:** Demographic and variables information of the sample.

Variable		N	M(SD)	Range (Min–Max)
Age	Total	26	23.59	19–28
			(2.00)	
	Male	10	23.6 (2.06)	19–28
	Female	16	23.5 (2.00)	19–27
Dyadic Decision Alignment Index (DDAI)	Congruent	4	-	-
	Divergent	10	-	-

**Table 2:** Statistics for non-significant results of theta, alpha, beta, and gamma bands.

EEG Band	Effect	*p*	*η^2^* *_p_*
Theta	ROI ^1^	0.999	0.000
	ROI * DDAI ^2^	0.907	0.009
Alpha	ROI ROI * DDAI	0.056	0.230
		0.359	0.089
Beta	ROI	0.954	0.004
	ROI * DDAI	0.887	0.011
Gamma	ROI ROI * DDAI	0.055	0.232
		0.263	0.114

^1^ ROI: Region of Interest; ^2^ DDAI: Dyadic Decision Alignment Index; * indicates an interaction effect

## Data Availability

The data presented in this study are available upon request from the corresponding author due to ethical reasons for sensitive personal data protection (requests will be evaluated according to the GDPR—Reg. EU 2016/679 and its ethical guidelines).

## Abbreviations

The following abbreviations are used in this manuscript:

EEG	Electroencephalography.
DRP	Dominant Reasoning Profile.
DDAI	Dyadic Decision Alignment Index.
MDT	Moral Deliberation Task.
ARI	Affective Rating Index.
CRI	Cognitive Rating Index.
ROI	Region of Interest.
EuDist	Euclidean Distance.
PSD	Power Spectral Density.
IFG	Inferior Frontal Gyrus.
DLPFC	Dorsolateral Prefrontal Cortex.
VMPFC	Ventromedial Prefrontal Cortex.
TPJ	Temporoparietal Junction.
fNIRS	Functional Near-Infrared Spectroscopy.
